# Genetic Dissection of Resistance to Gray Leaf Spot by Combining Genome-Wide Association, Linkage Mapping, and Genomic Prediction in Tropical Maize Germplasm

**DOI:** 10.3389/fpls.2020.572027

**Published:** 2020-11-02

**Authors:** Maguta Kibe, Sudha K. Nair, Biswanath Das, Jumbo M. Bright, Dan Makumbi, Johnson Kinyua, L. M. Suresh, Yoseph Beyene, Michael S. Olsen, Boddupalli M. Prasanna, Manje Gowda

**Affiliations:** ^1^International Maize and Wheat Improvement Center, Nairobi, Kenya; ^2^Jomo Kenyatta University of Agriculture and Technology, Nairobi, Kenya; ^3^International Maize and Wheat Improvement Center, Hyderabad, India

**Keywords:** GLS, GP, GWAS, SNP, disease resistance, JLAM

## Abstract

Gray leaf spot (GLS) is one of the major maize foliar diseases in sub-Saharan Africa. Resistance to GLS is controlled by multiple genes with additive effect and is influenced by both genotype and environment. The objectives of the study were to dissect the genetic architecture of GLS resistance through linkage mapping and genome-wide association study (GWAS) and assessing the potential of genomic prediction (GP). We used both biparental populations and an association mapping panel of 410 diverse tropical/subtropical inbred lines that were genotyped using genotype by sequencing. Phenotypic evaluation in two to four environments revealed significant genotypic variation and moderate to high heritability estimates ranging from 0.43 to 0.69. GLS was negatively and significantly correlated with grain yield, anthesis date, and plant height. Linkage mapping in five populations revealed 22 quantitative trait loci (QTLs) for GLS resistance. A QTL on chromosome 7 (*qGLS7-105*) is a major-effect QTL that explained 28.2% of phenotypic variance. Together, all the detected QTLs explained 10.50, 49.70, 23.67, 18.05, and 28.71% of phenotypic variance in doubled haploid (DH) populations 1, 2, 3, and F_3_ populations 4 and 5, respectively. Joint linkage association mapping across three DH populations detected 14 QTLs that individually explained 0.10–15.7% of phenotypic variance. GWAS revealed 10 significantly (*p* < 9.5 × 10^–6^) associated SNPs distributed on chromosomes 1, 2, 6, 7, and 8, which individually explained 6–8% of phenotypic variance. A set of nine candidate genes co-located or in physical proximity to the significant SNPs with roles in plant defense against pathogens were identified. GP revealed low to moderate prediction correlations of 0.39, 0.37, 0.56, 0.30, 0.29, and 0.38 for within IMAS association panel, DH pop1, DH pop2, DH pop3, F_3_ pop4, and F_3_ po5, respectively, and accuracy was increased substantially to 0.84 for prediction across three DH populations. When the diversity panel was used as training set to predict the accuracy of GLS resistance in biparental population, there was 20–50% reduction compared to prediction within populations. Overall, the study revealed that resistance to GLS is quantitative in nature and is controlled by many loci with a few major and many minor effects. The SNPs/QTLs identified by GWAS and linkage mapping can be potential targets in improving GLS resistance in breeding programs, while GP further consolidates the development of high GLS-resistant lines by incorporating most of the major- and minor-effect genes.

## Introduction

Maize is the most important cereal crop in sub-Saharan Africa (SSA), where more than 80% of the population rely on it as a source of food, income, and livelihood (Prasanna et al., 2020). Gray leaf spot (GLS) is a one of the major foliar diseases of maize caused by the polycyclic pathogens *Cercospora zeae-maydis* and *Cercospora zeina* (Crous et al., 2006; He et al., 2018). In eastern Africa, *C. zeae-maydis* is more prevalent. GLS poses a serious problem to maize production with estimated yield losses of more than 70% (Liu et al., 2016) under favorable conditions. The disease caused severe economic losses in SSA (Ward, 1996; Vivek et al., 2010; Kibata et al., 2011; Bekeko et al., 2018; Yigrem and Yohannes, 2019). Therefore, maize breeding programs in SSA typically incorporate GLS resistance in product pipelines.

Diagnostic symptoms of GLS include necrotic and chlorotic spots that run parallel to the leaf veins, rectangular fleck-type lesions; later-stage infection leads to severe blighting of leaves, stalk rotting, severe lodging, and premature death (Latterell and Rossi, 1983). Several factors contribute to the prevalence of GLS including conducive environment for disease development, monoculture of maize, and adoption of conservation tillage, which allows fungal inoculum to build up on crop residues. Moderate to high temperatures coupled with prolonged periods of high relative humidity also favor the development of disease symptoms (Ward, 1996). Chemical control has been recommended to combat GLS, but the application of fungicides is not economical especially for small and marginal farmers, and is also hazardous to human health with negative impacts on environment (Dhami et al., 2015). Breeding for resistant germplasm through conventional methods and by integrating advanced molecular tools is the most effective method to control diseases and to ensure maize-based food security in SSA.

Quantitative trait loci (QTLs) underlying resistance to several diseases in maize have been identified in the last two decades (Welz and Geiger, 2000; Shi et al., 2014; Xu et al., 2014; de Jong et al., 2018; He et al., 2018; Sitonik et al., 2019; Du et al., 2020; Lv et al., 2020). Despite the substantial number of QTLs reported, majority of them had huge confidence intervals, which represented large segments of chromosomes. In many cases flanking markers are very far from the causative mutations that can be easily lost during meiotic recombination and consequently limit their usefulness in breeding applications. Use of small mapping populations with low mapping resolution is also another major reason for the failure to identify robust and reliable markers associated with disease resistance. Although a biparental population-based genetic mapping approach offers high QTL detection power, the resolution remains low (Holland, 2007). In recent QTL mapping studies, the size of the mapping populations usually ranged between 100 and 300 individuals (Lehmensiek et al., 2001; Zwonitzer et al., 2010; Berger et al., 2014; He et al., 2018).

Genome-wide association studies (GWAS) have shown great potential by detecting QTL with high resolution, besides faster and accurate determination of recombination breakpoints (Zhu et al., 2008), but the detection power is fairly low and the false-positive rate is often high (Mammadov et al., 2015). Nevertheless, GWAS has been used successfully to identify QTL or genomic regions for some major diseases in maize at the whole-genome level, including maize lethal necrosis (MLN) (Gowda et al., 2015; Sitonik et al., 2019; Nyaga et al., 2020) and tar spot complex (Mahuku et al., 2015). GWAS of GLS was reported earlier in temperate maize germplasm (Benson et al., 2015; Mammadov et al., 2015) where significant SNPs associated with GLS resistance have been identified using diverse panels. However, GWAS for GLS resistance in tropical maize germplasm adapted to SSA agroecology was not reported so far.

Molecular marker-assisted breeding for improving disease resistance in maize is implemented in a few cases where QTLs with major effects were identified and validated; minor-effect QTLs are not part of the selection process, as these are often not consistent across different genetic backgrounds (Kuki et al., 2018). Genomic prediction (GP) is a newer approach that estimates the effects of all markers simultaneously, while omitting the stringent significance testing needed to identify QTL (Meuwissen et al., 2001). For GP, genetic markers spanning the whole genome are used with the assumption that all QTLs will be in linkage disequilibrium (LD) with at least one of these markers (Heffner et al., 2010). GP combines phenotypic and genotypic data of the training population to obtain genomic estimated breeding values (GEBVs) of the testing population that has been genotyped (Crossa et al., 2017; Wang et al., 2018). GP captures even the small effect QTLs/genes that are not detected by marker-assisted selection (Hayes et al., 2009).

In GP, the number of markers tend to be higher than the number of phenotypic observations; to account for this, GP applies various algorithms and models including genomic best linear unbiased prediction (GBLUP), rr-BLUP (Ridge regression), and Bayes (Crossa et al., 2017). GP reduces the time required for variety development and the cost per cycle as compared to phenotypic selection (Crossa et al., 2017; Beyene et al., 2019). However, applying GP in an association mapping population shows more effectiveness for traits possessing high heritability (Combs and Bernardo, 2013).

With this background, the objectives of the study were as follows: (1) to phenotypically characterize a genetically diverse association mapping panel and biparental populations for their responses to GLS, including correlation with other agronomic traits; (2) to conduct population-based QTL mapping and joint linkage association mapping (JLAM); (3) to identify marker-trait associations for GLS resistance through GWAS; and (4) to assess the usefulness of GP in breeding for GLS resistance in tropical maize.

## Materials and Methods

### Plant Materials and Field Trials

An association mapping panel developed at the international Maize and Wheat Improvement Center (CIMMYT), called the Improved Maize for African soils (IMAS) panel (Ertiro et al., 2020a), three doubled haploid (DH) populations [pop1 CML 550 x CML494 (107 lines); pop2 CML550 x CML504 (211 lines); pop3 CML550 x CML511 (107 lines)] (Ertiro et al., 2020b), and two F_3_ populations [pop4 CZL0618 x LaPostaSeqC7-F71-1-2-1-1B (183 lines); and pop5 CZL074 x LaPostaSeqC7-F103-1-2-1-1B (172 lines)] (Semagn et al., 2012; Zhang et al., 2015) were used in this study (Supplementary Table S1). The IMAS panel, comprising 410 CIMMYT maize (sub)tropical inbred lines, was used to evaluate the genetic architecture of resistance to several major diseases through GWAS (Gowda et al., 2015; Sitonik et al., 2019). The IMAS panel included lines adapted to tropical lowlands, African mid-elevation/subtropical, and the tropical highlands. All three DH populations used in this study were also used in earlier studies on MLN and low N stress conditions (Sitonik et al., 2019; Ertiro et al., 2020b); the two F_3_ populations were used to study the grain yield under optimum and drought stress conditions (Semagn et al., 2012; Zhang et al., 2015). The IMAS panel was evaluated in four location–year combinations [Kakamega (0°17′3.19″ N 34°45′8.24″ E, 1535 masl) and Kitale (1.0191° N 35.0023° E, 1900 masl) in 2013 and 2014]. DHpop2 and DH pop3 were evaluated in Kakamega and Kitale for 2 years in 2014 and 2015. DH pop1 was evaluated only in 2015 in the same two locations. F_3_ pop4 and F_3_ pop5 were evaluated in two locations in Kakamega and Embu (0°31′52″ S 37°27′02″ E, 1406 masl) at 2011 in Kenya (Supplementary Table S1). The list of the inbred lines, DH lines and F3 populations, and their phenotypic and marker data is available for all in CIMMYT depository^1^.

All the genotypes from IMAS panel, DH, and F_3_ populations were planted in 4-m-long single row plots in an alpha lattice design with two replications in independent trials at locations mentioned above. Two seeds were planted per hill and thinned to a single plant per hill, 3 weeks after emergence to ensure uniform plant density. Standard agronomic practices were followed. The chosen locations were natural hotspots for GLS; uniform disease infection across the trials at each location was observed. The IMAS panel, and the DH and F_3_ populations were evaluated for their responses to GLS in two to four environments (Supplementary Table S1). GLS disease severity is typically at its peak between the growth stages of tasseling and physiological maturity; therefore, disease severity data were recorded at the mid-silking and hard dough stages, and scored plot-wise on an ordinal scale of 1 (highly resistant, without disease symptoms) to 9 (highly susceptible, leading to necrosis). On the IMAS panel, in addition to GLS severity scoring, data were also collected for several other agronomic traits, including anthesis date (AD), anthesis-silking interval (ASI), plant height (PH), ear height (EH), ear position (EPO), ears per plant (EPP), husk cover (HC), ear rot (ER), corn rust (PS), Turcicum leaf blight (TLB), grain yield (GY), and grain moisture (MOI).

### Phenotypic Data Analyses

Since the phenotypic data for GLS was recorded based on an ordinal scale, we evaluated whether the data met the assumptions of the applied statistical model, i.e., normally distributed, constant variance, and independent (Rawlings et al., 1998). The analysis revealed that the GLS data met all the assumptions. For every population, each location–year combination was treated as an independent environment that resulted in four environments for the IMAS panel, DH pop2, and DH pop3, whereas the number of environments was two for DH pop1, F_3_ pop4, and F_3_ pop5. Analysis of variance for individual and across environments was undertaken using the ASREML-R (Gilmour et al., 2009) for each biparental population and the IMAS panel. The following statistical model was used to estimate variance components:

$${\text{Y}}_{\mathrm{ijko}}=\mu +{\text{G}}_{i}+{\text{E}}_{j}+{\left(\text{GE}\right)}_{\mathrm{ij}}+\text{R}{\left(\text{E}\right)}_{\mathrm{kj}}+{\text{B}(\text{R.E})}_{\mathrm{ojk}}+{\text{e}}_{\mathrm{ijko}},$$

where Y*_*ijko*_* is the phenotypic performance of the *i*th genotype at the *j*th environment in the *k*th replication of the *o*th incomplete block, *μ* is an intercept term, *G*_*i*_ is the genetic effect of the *i*th genotype, *E*_*j*_ is the effect of the *j*th environment, (*GE*)*_*ij*_* is the interaction effect between genotype and environment, *R(E*)*_*kj*_* is the effect of the *k*th replication at the *j*th environment, *B(R.E*)*_*ojk*_* is the effect of the *o*th incomplete block in the *k*th replication at the *j*th environment, and e*_*ijko*_* is the residual. The genotypic effect (*G*_*i*_), genotype by environment interaction, and effect of incomplete blocks were treated as random effects in order to estimate their variances and residual error variance. Environments and replications were treated as fixed effects. Assuming fixed genotypic effects, a mixed linear model (MLM) was fitted to obtain the best linear unbiased estimates (BLUEs). With ASREML-R, the significance of variance components were tested by model comparison (full model vs. half model) with likelihood ratio tests in which the halved *P* values were used as an approximation. Heritability (*H*^2^) was estimated as the ratio of genotypic to phenotypic variance components.

Broad-sense heritability (*H*^2^) was calculated for all the traits using the following equation:

$$H^{2}=\frac{\sigma_{g}^{2}}{\sigma_{g}^{2}+\frac{\sigma_{gl}^{2}}{l}+\frac{\sigma_{\varepsilon }^{2}}{lr}}$$

where σ^2^*_*g*_* is the genotype variance, σ^2^*_*gl*_* is the genotype × environment interaction variance, and σ^2^_ε_ is the error variance, with *l* representing number of environments and *r* denoting number of replications. META-R software (Alvarado et al., 2015) was used to obtain best linear unbiased prediction (BLUP) for each genotype across environments.

### Genotypic Data and Linkage Mapping

More detailed explanation on the molecular markers used and the linkage map construction for DH and F_3_ populations are available in earlier studies (Semagn et al., 2012; Sitonik et al., 2019; Ertiro et al., 2020b). In brief, the DNA of all inbred lines of the IMAS AM panel and the biparental populations was extracted from seedlings at 3–4 leaf stages and genotyped using the GBS platform at the Institute for Genomic Diversity, Cornell University, Ithaca, United States, using high-density markers, as per the procedure described by Elshire et al. (2011). For all three DH populations, TASSEL ver5.2 (Bradbury et al., 2007) was used to exclude SNPs with a heterozygosity of >5%. Whereas for all the five biparental populations, a minor allele frequency (MAF) of <0.05 and a minimum count of 90% were excluded by filtering from raw GBS SNP markers. Further, for each population, markers that are homozygous for both the parents and polymorphic between the parents were retained. Finally, SNPs were further filtered based on minimum distance between the markers. We used the criteria of minimum distance between adjacent SNPs as ≥200 kilobase pairs (kbps) to ensure uniform distribution of markers throughout the genome. For JLAM, markers from all three DH pops were combined, and markers with <1% missing value and >5% MAF and heterozygosity of <5% were retained. Finally, a set of 7490 SNPs that are uniformly distributed across the genome was used for JLAM analyses.

QTL IciMapping version 4.1 (Meng et al., 2015) was used to construct the linkage map based on data from all five biparental populations. QTL IciMapping was used to remove the highly correlated SNPs that do not provide any additional information by using an inbuilt tool BIN. This resulted in retention of 2105, 2699, 1962, 1130, and 1047 high-quality SNPs in DHpop1, pop2, pop3, F_3_pop4, and F_3_pop5, respectively. These SNPs were used to construct linkage maps using the MAP function, by selecting the most significant markers using stepwise regression. A likelihood ratio test was used to calculate the logarithm of odds (LOD) for each marker at a score of >3 with a 30-cM maximum distance between two loci. Three steps involved in constructing the linkage were grouping, ordering, and rippling. The Recombination Counting and ORDering (RECORD) algorithm was used to order markers. Grouping was done at LOD score > 3.0, and Sum of adjacent criterion (SAD) ripple was used to confirm marker order. The Kosambi mapping function (Kosambi, 1944) was used to transform the recombination frequencies between two linked loci. BLUPs across environments were used to detect QTLs based on Inclusive interval mapping (ICIM) for each population. Phenotypic variation explained by individual QTL and total variation explained by QTLs were estimated. QTL naming was done with letter “q” indicating QTL, followed by abbreviation of trait name, the chromosome, and marker position, respectively.

### Joint Linkage Association Mapping

For JLAM, high-quality, uniformly distributed 7490 SNPs across three DH populations were selected. The SNPs were then used to construct a linkage map based on their physical positions. A biometric model (Würschum et al., 2012) was used to perform JLAM, with BLUPs across environments and population being applied for analysis. After testing several biometric models, one that performed well for association studies in multiple biparental populations (Würschum et al., 2012) was used to conduct the JLAM. This model controls the differences in population means by incorporating population effect and the genetic background by using cofactors and marker effects across populations. This model was explained in detail by Liu et al. (2011) and Würschum et al. (2012). In brief, with this model, as a first step, cofactors were selected based on the Schwarz Bayesian Criterion (SBC, Schwarz, 1978) by including a population effect, and in the second step, *P-*values were calculated for the *F*-test by using a full model (including SNP effect) versus a reduced model (without SNP effect). Cofactors were selected by using PROC GLM SELECT from SAS 9.4 (SAS Institute Inc, 2015) and genome-wide scans for QTLs were applied in R version 3.2.5 (R Core Team, 2015).

### Genome-Wide Association Analyses

TASSEL ver5.2 (Bradbury et al., 2007) was used for GWAS. SNPs with a heterozygosity of <5%, a MAF of >0.05, and a minimum count of 90% were included by filtering from raw GBS data sets, and 337,110 high-quality SNPs were retained for further analysis in the IMAS AM panel. Distribution of these 337,110 GBS markers in the maize genome is presented with different color keys in the Supplementary Figure S1. BLUPs across environments were used as phenotypes in association mapping scans. Principal component analysis (PCA) was performed using TASSEL ver5.2. The principal components were used to correct for population structure and to create a two-dimensional plot to enable visualization of the probable population structure. A MLM that computes both PCs and kinship matrix (K) was applied for GWAS to correct for population structure (Yu and Buckler, 2006). The kinship matrix was calculated in Tassel ver 5.2 using a normalized Identity by State (IBS) option. The extent of LD of the genome was based on physical distances between the SNPs and the adjacent pairwise *r*^2^ values between high-quality SNPs from GBS (Remington et al., 2001). The “nlin” function in R was used to fit non-linear models into the genome-wide LD data by incorporating *r*^2^ as responses (*y* axis) and pairwise distances (*x* axis) as predictors. The average estimator for LD decay was calculated at a “significance” threshold of *r*^2^ = 0.1 and *r*^2^ = 0.2 cutoff points in relation to distance (Hill and Weir, 1988), and a representative scatter plot was drawn as LD between adjacent markers versus chromosome distance (kb). In this study, we used a MAF of 0.05 to assess LD as was applied in other studies (Li et al., 2014; Vos et al., 2017).

Scans for genome-wide marker-trait associations were carried out to detect main effect QTLs. *R*^2^ statistics was used to assess the amount of phenotypic variation explained by the model by simultaneously fitting all significant SNPs in a linear model. Multiple testing correction was performed to determine the significance threshold, where instead of 337,110 independent tests, the total number of tests was estimated based on the average extent of LD at *r*^2^ = 0.1 (Cui et al., 2016). With respect to the above, significant associations were declared when *P*-values in independent tests were less than 9.5 × 10^–6^. Candidate genes containing or being adjacent to the significant SNPs were obtained from the B73 gene set (version 2.0) in Maize GDB. BLAST searches were performed with 50-bp source sequences of the significantly associated SNPs against the “B73” RefGen_v2^2^.

### Genomic Prediction

RR-BLUP was used to carry out GP using a fivefold cross-validation (Zhao et al., 2011). BLUEs across environments for each of the biparental populations and across three DH populations were used for the analysis. For all biparental populations and IMAS panel, the same set of high-quality uniformly distributed 4000 SNPs with no missing values and MAF > 0.05 were used. Approaches used for GP include the “within population” approach in which individual biparental population and IMAS set were sampled to form a training and prediction set, a “joint population” or combined population prediction approach where data from three DH populations were combined and sampled randomly to form a testing and training set, and “across population” prediction in which IMAS association panel was used as a training set and each DH population was used as a testing set. For each approach, 100 iterations were done for sampling of the training and validation sets. RR-BLUP was implemented using the statistical software R (R Core Team, 2015).

## Results

The IMAS panel with a set of 410 lines and five biparental populations were evaluated against GLS in Kenya in two to four environments. GLS disease severity scores indicated comparable disease pressures across the tested environments as indicated by significant genotypic variance at each environment for each population (Supplementary Table S1). Further, significant (*p* < 0.05) Pearson correlations were also observed among phenotypic values determined at different environments for each population (Supplementary Table S2). This suggested that there was enough GLS disease pressure in each environment and the combined analysis across environments was not biased. The significant environmental variations (data not shown) observed for GLS indicated that the environments were distinct and provided unique information on the individual lines in the linkage and association mapping population.

**FIGURE 1:**
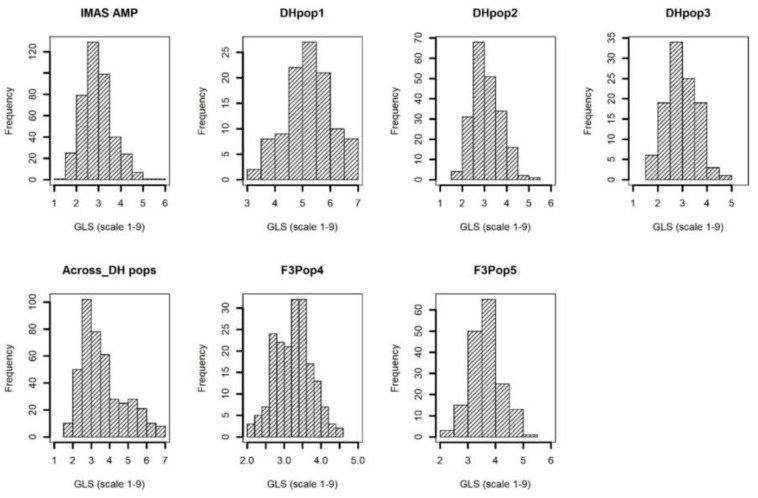
Phenotypic distribution of GLS disease severity in IMAS association mapping panel, three DH populations, across DH populations, and two F_3_ populations evaluated in two to four environments.

The disease severity was high in each of the environments as well as across environments, with the susceptible checks CLYN265 and DTPYC9-F13-2-3-1-2-B showing a score of 7 on the 1–9 scale. The frequency of the phenotypic values followed a near-normal distribution for individual populations and combined DH populations as well as IMAS panel (Figure 1). The IMAS panel, on average, was moderately resistant to GLS with a mean of 3.98, assessed on the 1–9 scale. Four parental lines of DH populations, CML494, CML504, CML511, and CML550, recorded GLS scores of 3.77, 3.39, 3.31, and 4.94, respectively. The DH populations developed from the four parents were moderately resistant with mean GLS scores of 5.24, 3.90, and 3.29 in DH pop1, 2, and 3, respectively, while the GLS score in the combined DH populations across environments was 4.52. For the F_3_ populations, the parental lines LapostaSeq-C7-F71 and CZL074 were GLS-resistant with mean scores of 2.60 and 3.00, respectively, whereas the other two parents LapostaSeq-C7-F108 and CZL618 had mean scores of 3.58 and 4.01, respectively. F_3_ pop4 and 5 recorded mean GLS scores of 3.24 and 3.84, respectively. Thus, significant variation for GLS disease severity was observed in all populations.

**FIGURE 2:**
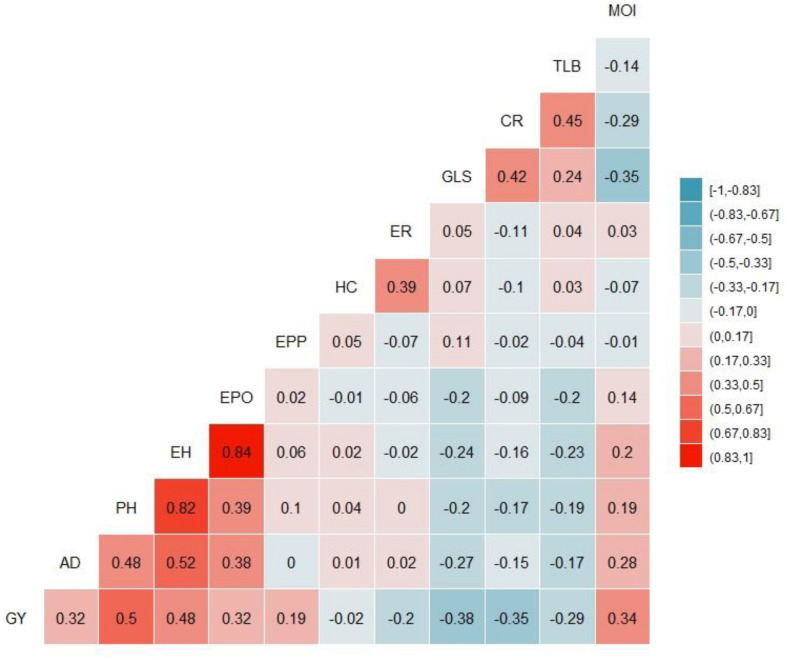
Phenotypic correlations among 12 traits evaluated in four environments. The correlation level is color-coded according to the color key plotted on the extreme right. Correlations with >0.12, and >0.15 were significant at 0.05 and 0.01 levels, respectively. GY, grain yield; *AD*, days to anthesis; *PH*, plant height; *EH*, ear height; *EPO*, ear position; *EPP*, number of ears per plant; HC, husk cover; GLS, gray leaf spot; TLB, Turcicum leaf blight; *CR*, corn rust; *MOI*, grain moisture content.

ANOVA, calculated across environments, revealed significant genotypic and genotype by environment (G × E) interaction variance in the IMAS panel (Table 1). Individual biparental populations and the combined DH populations had significant genotypic and G × E interaction variance except for F_3_ pop5 where the G × E interaction variance was not significant. Heritability estimates were moderate, ranging from 0.43 to 0.68. F_3_ pop4 had the lowest heritability estimates while DH pop2 had the highest estimates of heritability. The IMAS panel and combined DH population had heritability estimates of 0.56. Each population displayed adequate disease expression for both susceptible and resistant lines for response to GLS. The IMAS panel used in this study showed significant variations for all other agronomic traits including plant height, grain yield, ear height, and ear position (data not shown). GLS is negatively and significantly correlated with grain yield, anthesis date, ear position, grain moisture, plant height, and ear height (Figure 2). Correlation among GLS and anthesis date was significant, but low magnitude (-0.27), showing that flowering time is not a cofound effect in GLS resistance. Of the traits surveyed, the largest positive correlation was estimated between ear height and ear position (*r* = 0.84, *p* < 0.01) and between ear height and plant height (*r* = 0.82, *p* < 0.01).

**TABLE 1:** Means and components of variance for gray leaf spot disease severity for maize inbred lines from IMAS association panel, three DH and two F_3_ populations, as well as across DH populations evaluated in two to four environments.

Population	Mean	σ^2^_*G*_	σ^2^_*G* × E_	σ^2^_*e*_	h^2^	LSD	CV
IMAS AM panel	3.98	0.05**	0.08**	0.19	0.56	0.26	22.05
CML550XCML494 DHpop1	5.24	0.07**	0.04*	0.24	0.45	0.39	15.66
CML550XCML504 DHpop2	3.90	0.07**	0.02*	0.20	0.68	0.29	22.04
CML550XCML511 DHpop3	3.29	0.05**	0.03*	0.24	0.59	0.30	24.51
CZL618XLaPostaSeqC7-F71-1-2-1-1 F_3_pop4	3.24	0.03*	0.03*	0.10	0.43	0.11	24.36
CZL074XLaPostaSeqC7-F103-1-2-1-1 F_3_pop5	3.70	0.04*	0.01	0.12	0.53	0.26	23.23
Across three DH populations	4.62	0.19**	0.13**	0.33	0.56	0.53	24.80

The environment-wise statistics are given in Supplementary Table S1. *, ** Significance at P < 0.05 and P < 0.01, respectively. σ^2^_G_, genotypic variance; σ^2^_G × E_, genotypic × environment interaction variance; σ^2^_e_, error variance; h^2^, broad sense heritability.

Population structure was diagnosed using STRUCTURE software for K (number of clusters/fixed subgroups). Most of the significant change was observed when K was increased from one to two and one to four. Structure results of *K* = 4 was the best probable partition, as they showed high consistency with significant delta *K* values, geographical origin, and pedigree history (Supplementary Figure S2). PCA indicated a clear diverse population structure within the panel. The first two PCs, PC1 and PC2, explained a variation of 35.99 and 19.56%, respectively. Further, LD for the entire genome was calculated using 11,035 SNPs. LD decay of the panel was rapid with increase in physical distance. Physical distance at a cutoff value of *r*^2^ = 0.1 was found to be 14.97 kb and that at *r*^2^ = 0.2 was 5.23 kb (Supplementary Figure S3).

Linkage map for each of the populations was constructed. The number of progenies or lines, markers, map lengths, and average genetic distances between the markers for each biparental population are presented in Supplementary Table S3. Linkage mapping detected one QTL (*qGLS5-217*) for GLS in DH pop1 on chromosome 5, which explained 11% of the phenotypic variance (Table 2). Five QTLs were identified for DH pop2 on chromosomes 1, 5, 7, and two on chromosome 8, which individually explained phenotypic variance ranging from 1.5 to 28.2%, and together explained 49.7% of the total phenotypic variance. In DH pop3, only two QTLs were detected on chromosomes 1 and 10, which individually explained 16.6 and 8.7% of phenotypic variance, and together explained 23.7% total phenotypic variance. Seven QTLs were detected in F_3_ pop4, which individually explained 3.21–14.36% of phenotypic variance and together contributed for 18.05% of total phenotypic variance. In the F_3_ Pop5, seven QTLs were detected, distributed on chromosomes 1, 2, 4, 5, and 6, individually explaining 2.53–12.79% of phenotypic variance. The QTL on chromosome 7 (*qGLS7-105*) in DH pop2 was found to explain the largest proportion of phenotypic variance (28.2%). Overlapping of QTLs was compared based on the physical distance between the flanking markers, as the linkage maps were not comparable due to different sets of markers used. The QTL *qGLS1-104* on F_3_ pop5 overlapped with the *qGLS1-123* on F_3_ pop4, *qGLS1-155* in DH pop3, and *qGLS1-158* in DH pop2 (Table 2). Another QTL on chromosome 4 *qGLS4-190* in F_3_ pop4 overlapped with *qGLS4-157* detected in F_3_ pop5. QTL *qGLS5-217* on DH pop1 overlapped with *qGLS5-217* QTL detected on F_3_ pop5. The QTLs detected between 155 and 159 Mbp on chromosome 1 in DH pop2 and DH pop3 were in close physical proximity and could possibly represent the same QTL.

**TABLE 2:** Detection of QTL associated with resistance to GLS, their physical positions, and genetic effects in three DH populations and two F_3_ populations.

QTL name^a^	Chr	Position (cM)	LOD	PVE (%)	Add	Dom	Total PVE (%)	Flanking markers		Left CI (cM)	Right CI (cM)
**CML550xCML494 DHpop1**											
*qGLS5-217*	5	212	2.76	10.99	0.06	−	10.5	S5_201939197	S5_217052211	190.5	214.5
**CML550xCML504 DHpop2**											
*qGLS1-158*	1	244	3.34	1.52	0.04	−	49.7	S1_158718899	S1_158003946	242.5	244.5
*qGLS5-18*	5	488	4.12	1.91	0.05	−		S5_17891733	S5_19048161	485.5	489.5
*qGLS7-105*	7	266	40.22	28.2	0.17	−		S7_105221050	S7_113205468	264.5	266.5
*qGLS8-108*	8	245	3.28	2.23	0.05	−		S8_107791296	S8_148484309	235.5	258.5
*qGLS8-8*	8	406	11.92	6.02	0.08	−		S8_8273703	S8_9875577	402.5	410.5
**CML550xCML511 DHpop3**											
*qGLS1-155*	1	417	4.86	16.6	0.07	−	23.67	S1_154764228	S1_157179343	415.5	417.5
*qGLS10-97*	10	181	2.7	8.73	0.05	−		S10_96772644	S10_99957462	179.5	181.5
**CZL0618xLaPostaSeqC7-F71-1-2-1-1B F_3_ pop4**											
*qGLS1-123*	1	568	4.66	5.70	–0.39	–0.13	18.05	**S1_122885347**	**S1_123767518**	567.5	569.5
*qGLS3-26*	3	32	3.16	3.81	–0.31	0.08		S3_33059091	S3_26022906	25.5	34.5
*qGLS4-190*	4	192	2.60	14.36	0.44	–0.83		**S4_4754149**	**S4_192217274**	188.5	198.5
*qGLS4-204*	4	330	2.58	3.21	0.13	–0.39		S4_203557175	S4_224911596	326.5	333.5
*qGLS4-189*	4	359	2.71	3.29	0.26	–0.20		S4_187296983	S4_189157835	350.5	363.5
*qGLS4-187*	4	370	3.16	3.94	–0.27	–0.30		S4_175739782	S4_187296983	367.5	372.5
*qGLS9-143*	9	453	3.36	4.19	0.33	0.12		S9_143037428	S9_143894887	447.5	458.5
**CZL074xLaPostaSeqC7-F103-1-2-1-1B F_3_ pop5**											
*qGLS1-281*	1	42	2.52	8.01	–0.55	0.08	28.71	S1_281168712	S1_285979058	40.5	43.5
*qGLS1-104*	1	510	2.68	12.79	–0.55	–0.26		**S1_103385029**	**S1_241404317**	508.5	510.5
*qGLS2-37*	2	444	3.54	3.36	0.24	–0.42		S2_37133478	S2_43353313	442.5	446.5
*qGLS4-157*	4	304	7.97	6.26	0.42	0.18		**S4_93244319**	**S4_157631782**	299.5	308.5
*qGLS5-217*	5	176	2.92	3.47	–0.36	0.00		**S5_208066400**	**S5_217416666**	167.5	182.5
*qGLS5-52*	5	376	2.64	7.19	–0.34	1.06		S5_51355494	S5_131040408	375.5	378.5
*qGLS6-69*	6	90	2.81	2.53	–0.57	–0.25		S6_68207704	S6_110436702	87.5	93.5

*^a^QTL name composed by the trait code followed by the chromosome number in which the QTL was mapped and the physical position of the flanking markers in Mb. Chr, chromosome; LOD, logarithm of odds; Add, additive effect; Dom, dominance effect; PVE, phenotypic variance explained. Markers with bold letters are the QTL consistent across at least two populations. The marker name indicates the chromosome number followed by its physical position; for example, S6_68207704 denotes that the marker is in chromosome 6 at a position of 68,207,704 bp (Ref Gen_v2 of B73). CI, confidence interval.*

Joint linkage association mapping analyses revealed 14 QTLs distributed across six chromosomes, 3 QTLs on chromosome 1, 5 QTLs on chromosome 5, 2 QTLs each on chromosomes 4 and 8, and 1 QTL each on chromosomes 6 and 9. These QTLs individually explained 0.1–15.7% of the phenotypic variance (Table 3). The QTL on chromosome 4 (*qGLS4-163*) had the largest effect at 15.7% of phenotypic variation and was found overlapping with the QTL *qGLS4-190* detected on F_3_ pop4 (Tables 2, 3). In addition, *qGLS1-158* overlapped with the QTL *qGLS1-104* detected in F_3_ pop5 and *qGLS1-158* in DH pop2. Another QTL, *qGLS4-154*, detected in JLAM also overlapped with QTL *qGLS4-190* in F_3_ pop4 and *qGLS4-157* in F_3_ pop5. A QTL on chromosome 5, *qGLS5-19*, overlapped with QTL *qGLS5-18* detected on DH pop2. Two QTLs detected in JLAM *qGLS5-213* and *qGLS5-216* were falling within the confidence interval of the QTL *qGLS5-217* detected in DH pop1 and F_3_ pop5 (Tables 2, 3). Large variation was observed for both allele substitution effects (α effect) even with changing in signs and QTL effects (Table 3).

**TABLE 3:** Analysis of GLS trait-associated markers, allele substitution (α) effects, and the total phenotypic variance (*R*^2^) of the joint linkage association mapping based on combined data from three DH populations.

Marker	QTL name^a^	chr	Position (Mbp)	α-effect	*P*-value	PVE (%)
S1_15169935	*qGLS1-15*	1	15.17	–0.04	2.05E-01	0.3
S1_56220954	*qGLS1-56*	1	56.22	0.11	6.14E-04	2.1
S1_158003964	*qGLS1-158*	1	158.00	–0.11	5.21E-05	2.9
S4_154047619	*qGLS4-154*	4	154.05	0.02	4.83E-01	0.1
S4_163681762	*qGLS4-163*	4	163.68	0.39	2.45E-19	15.7
S5_7702493	*qGLS5-7*	5	7.70	0.05	3.69E-02	0.5
S5_13738035	*qGLS5-13*	5	13.73	0.14	2.92E-01	0.1
S5_19525108	*qGLS5-19*	5	19.52	–0.13	7.61E-08	3.7
S5_212619482	*qGLS5-213*	5	212.62	–0.16	6.08E-02	0.4
S5_215811751	*qGLS5-216*	5	215.81	0.04	1.82E-01	0.2
S6_116334713	*qGLS6-116*	6	116.34	–0.34	3.35E-03	1.1
S8_13783996	*qGLS8-14*	8	13.78	–0.19	2.34E-11	5.9
S8_166561872	*qGLS8-166*	8	166.56	0.42	1.07E-17	10.0
S9_79119948	*qGLS9-79*	9	79.12	–0.03	2.39E-01	0.2

*^a^QTL name composed by the trait code followed by the chromosome number in which the QTL was mapped and a physical position of the QTL. Chr, chromosome; PVE, phenotypic variance explained. The marker name indicates the chromosome number followed by its physical position; for example, S6_68207704 denotes that the marker is in chromosome 6 at a position of 68,207,704 bp (Ref Gen_v2 of B73).*

**FIGURE 3:**
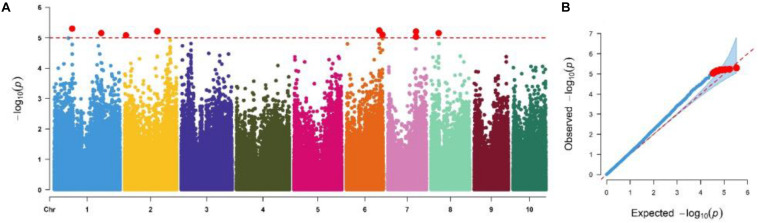
**(A)** Manhattan and quantile–quantile plots of a mixed linear model for GLS resistance in the IMAS association mapping panel evaluated in four environments. The dashed horizontal line depicts the significance threshold (*P* = 9.5 × 10^– 6^). The *X*-axis indicates the SNP location along the 10 chromosomes, with chromosomes separated by different colors; *Y*-axis is the -log10(*P* observed) for each analysis. **(B)** Quantile–quantile plots.

We performed GWAS based on MLM, which corrects for both the population structure and familial relatedness to avoid type I errors. Further, type II errors was avoided by observing the distribution of null *versus* alternative hypotheses in quantile–quantile (QQ) plots (Figure 3). GWAS analyses revealed the quantitative genetic nature of the GLS resistance and identified 10 marker-trait associations (MTA) in five chromosomes (Table 4) based on a significant threshold *p* value of 9.5 × 10^–6^. SNPs identified for GLS severity explained moderate phenotypic variation ranging from 6 to 9%. The SNP *S1_215340710* is co-located with QTL *qGLS1-104* detected in biparental F_3_ pop5 (Tables 2, 4). The B73 maize genome reference V2.0 sequence was used to identify putative candidate genes based on significant SNP associations. Sets of putative candidates were identified together with their predicted functions (Table 4).

**TABLE 4:** List of significant SNPs and candidate genes associated with GLS resistance in the IMAS association panel evaluated in four environments.

SNP name^a^	Chr	Position	MLM-P value	*R*^2^ (%)	MAF	Allele	MAE	Putative candidate genes	Predicted function of candidate gene
S1_82702920	1	82702920	5.0287E-06	0.06	0.46	G/A	0.09	GRMZM2G506660	ATP binding protein
S1_215340710	1	215340710	7.0783E-06	0.07	0.10	T/C	–0.06	GRMZM2G106558	myb146—MYB-transcription factor 146
S2_5924471	2	5924471	8.3283E-06	0.06	0.07	T/C	–0.7	GRMZM2G372074	Protein encodes a protease inhibitor/LTP family protein
S2_148656983	2	148656983	6.2212E-06	0.07	0.42	C/G	0.17	GRMZM2G115659	Carbohydrate transporter/sugar porter/transporter
S2_148656984	2	148656984	6.0999E-06	0.07	0.43	G/C	0.17	GRMZM2G115658	Carbohydrate transporter/sugar porter/transporter
S6_150800750	6	150800750	5.8341E-06	0.08	0.06	T/G	0.29	GRMZM2G067156	Unknown
S6_165317183	6	165317183	7.9948E-06	0.07	0.43	C/T	0.01	GRMZM2G092475	Probable sodium/metabolite cotransporter BASS4
S7_128373677	7	128373677	9.3869E-06	0.07	0.07	T/A	0.03	GRMZM2G416632	Glutathione transferase23
S7_128375218	7	128375218	6.2406E-06	0.08	0.05	T/C	0.32	GRMZM2G416625	Purine permease 3
S8_34664259	8	34664259	7.0625E-06	0.07	0.14	A/C	–0.05	GRMZM2G051522	Protein DOG1-like 4

*MAF, minor allele frequency; R^2^, proportion of phenotypic variance explained by SNP. ^a^The exact physical position of the SNP can be inferred from the marker’s name; for example, S1_82702920: chromosome 1; 82,702,920 bp (Ref Gen_v2 of B73).*

**FIGURE 4:**
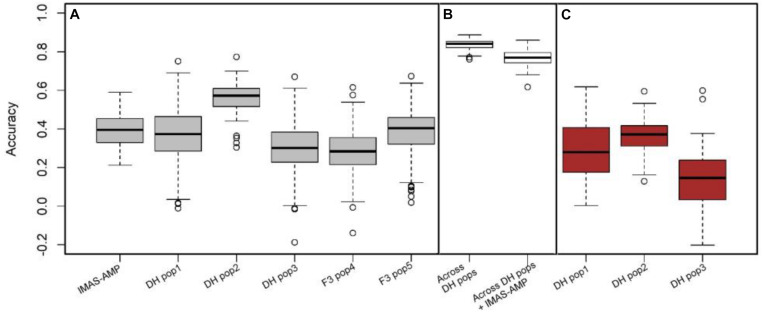
Genome-wide prediction accuracies for GLS resistance in biparental and IMAS AM panel based on three different scenarios. **(A)** Estimation and prediction within IMAS AM panel and biparental populations; **(B)** estimation and prediction combined DH populations and combined all DH populations and IMAS AM panel; **(C)** prediction of the DH population using IMAS AM panel as a training set with fivefold cross-validation.

The predictive ability of the GP model was determined as the correlation between GEBVs and observed phenotypes. The fivefold cross-validation revealed low to moderate prediction correlations of 0.39, 0.37, 0.56, 0.30, 0.29, and 0.38 for within IMAS association panel, DH pop1, DH pop2, DH pop3, F_3_ pop4, and F_3_ po5, respectively (Figure 4). The prediction correlations were increased to 0.84 when the prediction was based on combined three DH populations. Combining both IMAS association panel and all DH populations increased the diversity of the training set, but the correlation was still high at 0.77. In breeding, prediction of new population using one diverse panel as common training set is desired. However, this could affect the prediction accuracy, particularly for complex traits. Nevertheless, for GLS, using IMAS association panel as the training set, we predicted the response of each DH population and observed cross-validated correlations of 0.29, 0.36, and 0.14 for DH pop1, DH pop2, and DH pop3, respectively (Figure 4).

## Discussion

Gray leaf spot is an economically important foliar disease of maize in SSA. With the changing climates and ever-increasing maize monoculture in SSA, GLS becomes a more serious threat to maize production in the future. Understanding the genetic basis of GLS resistance is important to design an effective breeding strategy for developing and deploying GLS-resistant parental lines and hybrids. Breeding for GLS resistance is influenced by several factors including genotype, environment, and their interactions. The present study used CIMMYT’s genetically diverse tropical and subtropical maize breeding lines and populations, with the aim to identify genomic regions for GLS resistance and validate the same across different populations and earlier reports. Five biparental populations were used to detect the QTL for GLS resistance with high detection power, besides an association mapping panel to find marker-trait associations.

The phenotypic data for GLS disease severity score in each of the biparental populations as well as combined three DH populations and association mapping panel supported the quantitative nature for GLS resistance (Figure 1). Several previous studies using either biparental populations (Berger et al., 2014; Liu et al., 2016) or an association mapping panel (Mammadov et al., 2015; Kuki et al., 2018) indicated polygenic control of GLS resistance. We also observed significant genotypic variance and moderate heritabilities indicating good prospects for introgressing GLS resistance in breeding programs, similar to the observations in some earlier studies (Berger et al., 2014; Mammadov et al., 2015; Liu et al., 2016; Kuki et al., 2018).

Tropical and subtropical maize germplasm possess a huge amount of unexplored genetic diversity, which provides insights into resistance for several key traits used in breeding programs (Liu et al., 2016). Moderate to high heritability within the biparental populations for GLS resistance is reported to be controlled by additive gene action (Gordon et al., 2006). QTL analyses in five biparental populations revealed 22 potential QTLs providing resistance to GLS. Several QTLs were consistently detected at least in two populations; for example, QTL *qGLS1-104* on F_3_ pop5 overlapped with the QTLs on F_3_ pop4, DH pop2, and DH pop3. Another QTL *qGLS4-190* in F_3_ pop4 overlapped with QTL in F_3_ pop5, and QTL *qGLS5-217* in DH pop1 overlapped with QTL detected on F_3_ pop5 (Table 2). There were five QTLs (*qGLS1-104*, *qGLS1-155*, *qGLS4-190*, *qGLS5-217* and *qGLS7-105*) with major effects (>10% phenotypic variance explained) detected on chromosomes 1, 4, 5, and 7 in five biparental populations. A major QTL (*qGLS5-217*) identified in DH Pop1, explaining about 11% of total phenotypic variance and located between 201 and 207 Mbp, was consistent with a consensus QTL in bin 5.03 reported by Shi et al. (2007) in a meta-QTL study. A study carried out by Liu et al. (2016) also identified a major QTL on bin 5.04, which coincided with a similar QTL for flowering time and showed linkage between GLS resistance and flowering time. In the findings of QTL mapping within DH pop2, only the QTL on chromosome 7 between 105 and 113 Mbp, *qGLS7-105*, was found to have a significant effect (*R*^2^ of 28.3%). For this major QTL *qGLS7-105*, the segregation alleles from two tightly linked flanking SNPs reveal that DH lines and inbred lines from the IMAS panel with low DS scores were strongly associated with GLS-tolerant parent CML504 (Supplementary Figure S4). However, the efficiency of these flanking markers should be assessed further through KASP (Kompetitive allele-specific PCR) assays, where we can check these markers’ ability to identify GLS-resistant and susceptible genotypes. In the previous study carried out in South Africa, Berger et al. (2014) identified various QTL hotspots for GLS resistance in chromosomal bins 7.02 and 7.03. “Hotspot,” in this case, is a genomic region comprising multiple genes (clusters) that correspond to a particular trait or a group of related traits. Similar hotspots for GLS have been observed in various studies within chromosome 1 (Clements et al., 2000; Lehmensiek et al., 2001; Pozar et al., 2009; Berger et al., 2014; Lv et al., 2020). Our findings also suggest a possible hotspot within DH pop3 on chromosome 1; QTL *qGLS1-155* explained 16.6% phenotypic variation. The same case applies for two QTLs (*qGLS8-108* and *qGLS8-8*) on chromosome 8 within DH pop2, as was also reported by Bubeck et al. (1993) and Chung et al. (2011). Overall, several QTLs were consistent with the previous studies indicating their reliability to be used in applied breeding.

Joint linkage association mapping explores both within- and across-population variations that enabled the detection of novel QTL, which would have been omitted by linkage mapping. In line with this expectation, we found that 8 out of 14 QTLs detected were novel, not observed in linkage mapping. JLAM results indicated the influence of many genes with minor effects on GLS resistance, as shown by the many identified QTLs that explained low phenotypic variation. In general, linkage mapping confines a QTL to a 10–20 cM interval because of limited recombination events during development of mapping population. Identifying QTLs that are consistent across populations, fine mapping of these QTLs, and construction of a high-resolution linkage map could help identify markers for cloning the QTL. On the contrary, six QTLs detected through JLAM overlapped with the QTL detected in linkage mapping, revealing not only stability but also help in reducing the confidence interval of the QTLs and may even be closer to the causal variant responsible for GLS resistance.

Population structure is an essential aspect as it influences detected MTAs including the false positives in an association mapping panel. The present study used CIMMYT’s elite maize lines from multiple breeding programs, including Africa highland, mid-elevation, and lowland tropics. We observed a moderate population structure with PC1 and PC2 explaining 35.99 and 19.56% of variation, respectively (Supplementary Figure S2). The diversity panel lines did not cluster into a group but were scattered among different groups. Similar findings were also observed by George et al. (2004) who studied the diversity of Asian inbred lines including CIMMYT’s tropical and subtropical lines of the region and concluded that the lines had a significant genetic diversity.

The mapping resolution of GWAS and number of SNPs required for desired marker density are dependent on the magnitude of LD and LD decay with genetic distance (Myles et al., 2009). The correlation between alleles in different genomic locations is generally based on the historical recombination between polymorphisms and hence requires a large population for study. The genome-wide average LD decay was 14.97 kb at *r*^2^ = 0.1 and 5.23 kb at *r*^2^ = 0.2, similar to Rashid et al. (2018) observed in their association panel. LD decayed rapidly with distance between sites but showed substantial variation among loci. Previous studies indicated a rapid decay in tropical maize germplasm as compared to the temperate germplasm; the high LD decay in tropical maize suggests a broader genetic base, resulting from high recombination events (Lu et al., 2009). This provides breeders with an opportunity to select germplasm that integrates high grain yield with disease resistance and abiotic stress tolerance.

The ad hoc statistics ΔK was used to determine the optimum number of subgroups based on the output log likelihood of data [LnP (D)] of STRUCTURE. The peaks of the line plot (Supplementary Figure S2) suggest that the population could be divided into two or four distinct groups in order of possibility, with the *K* = 4 of delta *K* intersecting with LnP (D) showing higher possibility. At *K* = 2, the panel could be grouped into tropical and subtropical groups with group 2 (G2) occupying the bulk. When *K* = 4, G2 appeared as a mixed group and was further divided into three groups. These three groups could be divided into highland, lowland, and mid-elevation. The clustering of the population using STRUCTURE correlated highly with known pedigree information and origin of the lines.

Multiple studies have been carried out earlier to evaluate the genetic architecture of GLS resistance using both biparental populations and association mapping panels, especially using temperate maize germplasm. Berger et al. (2014), Shi et al. (2014), and Liu et al. (2016), used F2:3 families, association mapping panel, and F_7_ RILs, respectively, and identified several QTLs for GLS resistance. Detection of QTLs for resistance to GLS was found to be variable between seasons and locations (Bubeck et al., 1993; Lukman et al., 2012). The use of a large tropical association mapping panel and testing across multiple environments in the present study enabled us to identify putatively associated SNPs for GLS resistance. We identified 10 SNPs significantly associated with GLS each explaining between 6 and 8% of total phenotypic variation. This concurred with the polygenic nature of GLS resistance where many genes with minor effects control disease resistance (Asea et al., 2012). Comparing QTL positions between different studies was difficult due to the use of different populations, genetic maps, and statistical tests to define QTL; therefore, we have opted to use the maize core bin regions to compare the QTL (Wisser et al., 2006; Balint-Kurti et al., 2008). Three chromosomal bins were identified in this study: 8.03 with two significantly associated SNPs, and 7.03 and 1.05 were the same as those identified by Shi et al. (2014).

The most significant SNP for GLS was in proximity with the *GRMZM2G506660* gene in bin 1.05, which encodes for adenosine-5′-triphosphate (ATP) binding protein (ABP). ABPs contain a binding site for interaction with ATP, an energy molecule. This binding site forms a platform for conversion of ATP to adenosine diphosphate (ADP), realizing energy for use by the protein, or conforms to the protein to change shape and act as a catalytic enzyme. Many ADPs being transmembrane proteins are responsible for the transport of a wide variety of micro- and macromolecules across intra- and extracellular membranes. They have roles in cellular motility, membrane transport, and regulation of various metabolic processes. *GRMZM2G115658* on chromosome 2 bin 2.05 is a carbohydrate transporter gene, while *GRMZM2G092475* on chromosome 6 bin 6.07 is a sodium/metabolite co-transporter gene within the chloroplast. Chloroplast plays a crucial role in metabolism and energy supply to photosynthetic organisms. They possess transporters and channels within the thylakoid membranes and envelope, hence mediating exchange of metabolites and ions within the chloroplast stroma, cytosol, and different sub-compartments of the chloroplast. *GRMZM2G115658* is involved in the regulatory movement of carbohydrate in and out or within a cell through a pore or transporter, and in turn regulating the amount of energy within a cell.

With all these putative candidate genes identified based on associated SNPs, it is evident that transporter genes and channels are somehow involved in plant defense, well demonstrated by the RTM system in Arabidopsis (Chisholm et al., 2000; Gowda et al., 2015), also suggesting that plants can resist pathogens in multiple ways. Since plant pathogens need to move within and between cells through transporter channels for spreading infection, there is high probability that the putative candidate genes identified in this study are involved in disease resistance in maize. However, this warrants further research on the roles of various transport proteins before potential use in strategies for enhancing resistance to GLS.

*GRMZM2G372074* is a lipid transfer protein encoding gene on chromosome 2 bin 2.02 characterized by a hydrophobic cavity. It functions in the transfer of lipophilic compounds to cuticular surface of epidermal cells and is directly involved in plant defense against pathogens. Minimal amounts of glycophosphatidylinositol (GPI)-anchored lipid transfer proteins (LPTGs) increase susceptibility to penetration of epidermal cell by pathogens (Fahlberg et al., 2019). *GRMZM2G416632* on chromosome 7 bin 7.03 is a gene coding for glutathione transferase23 (GST), which are multifunctional enzymes, highly induced by a wide range of biotic stress factors. This enzyme was found to be up-regulated by early phase of fungal microbial infections. Silencing and overexpression of specific GSTs modify pathogen multiplication rates and subsequent amounts of pathogens in plants (Gullner et al., 2018).

GWAS and GP can be carried out simultaneously since both use the same set of markers and mapping populations, and it captures both minor- and major-effect QTLs (Chaikam et al., 2019; Sitonik et al., 2019). GP is helpful in accelerating the breeding cycle by facilitating the rapid selection of superior genotypes through ease in genotyping and availability of a wide range of markers, which capture maximum favorable alleles. The potential for different GS-based models in identifying lines with favorable traits in maize has been examined in various studies (Zhang et al., 2015; Crossa et al., 2017; Gowda et al., 2018; Beyene et al., 2019). Moderate to high accuracies observed in this study for the biparental populations and diversity panel offer promise in breeding for GLS resistance in tropical maize. Prediction accuracy of the diversity panel was in agreement with various studies on moderately complex traits like MLN (Gowda et al., 2015), maize chlorotic mottle virus (Sitonik et al., 2019), and northern corn leaf blight (Chung et al., 2011). According to Sitonik et al. (2019), significant genetic structure and high LD between adjacent markers of the diversity panel resulted in a moderate prediction accuracy, which could also be attributed to its moderate heritability. Combining the individual DH populations and applying cross-validations to obtain the training set and prediction set from the total DH population resulted in substantial improvement in the prediction accuracy (Figure 4). This was due to the increase in population size of the training set and high relatedness between training and prediction sets. Adding the GWAS panel to the combined DH populations increased the population size but resulted in a slight drop in the accuracy, which was due to the varying degrees of relatedness between the sets and increase in diversity of the training set.

The relative advantage of GP over phenotypic selection determines its routine use in breeding programs. This implementation is dependent on cost-effective and high-throughput genotyping technology like GBS, which provides a platform to genotype many maize lines at relatively low costs (Elshire et al., 2011). This study showed consistent results between GP and phenotypic selection accuracy indicating the possibility to integrate GP with phenotypic selection to improve the efficiency with less resources (Beyene et al., 2019). In terms of gain per year, GP is much more efficient taking into account the likelihood of completing three cycles per year (Lorenzana and Bernardo, 2009). Rapid decline in the cost of genotyping makes it possible to routinely apply GP in breeding. Combining GWAS and the predictive capabilities of GP will also improve the prediction accuracy by using information on major QTLs from GWAS or linkage mapping. Prediction accuracy dropped for the across- and within-population approach where the GWAS population was used as the training population, similar to Liu et al. (2018) and Sitonik et al. (2019) who used diversity panel or natural population to predict biparental populations. The prediction within each DH population revealed an accuracy of 0.37, 0.56, and 0.30 for DH pop1, 2, and 3, respectively, while predicting each biparental population by using the IMAS panel as training set showed 0.29, 0.36, and 0.14 accuracy for DH pop 1, 2 and 3, respectively. These correlations are low compared to prediction within populations. However, when the square root of heritability is compared to the GP correlations, they show that observed gain in phenotypic selection and GP were comparable. This relatively high prediction accuracy by using the IMAS panel as training set could be attributed to the populations’ relatedness to the diversity panel as all parents of the three DH populations were part of the GWAS mapping panel and are related to several lines derived from a (sub)tropical breeding program. The reduction in accuracy is also attributed to the magnitude of trait genotypic variability and heritability in each population. Nevertheless, the predicted accuracies are positive, and under the assumption of three cycles per year possibility, the total selection gain is comparable to phenotypic selection gain. The study thus shows promise for using a common training population or historical data to predict GLS resistance in several connected but independent populations.

## Conclusion

In this study, we used one association panel comprising 410 (sub)tropical maize inbred lines for GWAS and GP to understand the genetic basis of resistance to GLS. We also studied five biparental populations using linkage mapping and JLAM to understand the underlying architecture of the trait. Phenotypic correlations of studied traits indicated the potential use of these populations for selection of superior lines. Linkage mapping identified several minor- and major-effect QTLs with a few overlapping across populations, while many QTLs are population-specific for GLS resistance. GWAS scan revealed 10 SNPs associated with GLS resistance. The putative candidate genes identified in the study and their proposed functions require further validation to confirm the involvement of these genes in GLS resistance. Several QTLs identified in this study were found to be overlapping across different analyses, and with QTLs or associated SNPs reported earlier in temperate maize germplasm. These genomic regions can serve as potential selection targets to improve resistance to GLS. GP can be used within populations to predict the response of the germplasm to GLS resistance. Having a common training population comprising lines with diverse representation from a breeding program with good quality phenotypic data and genotyped with high-density markers holds promise in breeding for GLS resistance.

## Data Availability Statement

The datasets presented in this study can be found in online repositories at: https://data.cimmyt.org/dataset.xhtml; jsessionid=dc30e94cd6c7318ac8df044c42eb?persistentId=hdl%3 A11529%2F10548467&version=DRAFT. The names of the repository/repositories and accession number(s) can be found in the article/Supplementary Material.

## Author Contributions

MK, BD, DM, BP, SN, and MG conceived the experiments. BD, MG, MK, LS, and YB conducted the field evaluations and phenotyping. MG, SN, and JB coordinated the GBS experiments. MG and MK carried out the GWAS and GS analyses. MG, BD, DM, YB, MO, BP, MK, JB, LS, JK, and SN interpreted the results and drafted the manuscript. All the authors contributed to the article and approved the submitted version.

## Conflict of Interest

The authors declare that the research was conducted in the absence of any commercial or financial relationships that could be construed as a potential conflict of interest.

## Footnotes

1 https://data.cimmyt.org/dataset.xhtml;jsessionid=dc30e94cd6c7318ac8df044c42eb?persistentId=hdl%3A11529%2F10548467&version=DRAFT

2 https://www.maizegdb.org/gbrowse/maize_v2
