# Supplementary material for: Genetic Dissection of Resistance to Gray Leaf Spot by Combining Genome-Wide Association, Linkage Mapping, and Genomic Prediction in Tropical Maize Germplasm
Source: Front Plant Sci. 2020 Nov 2;11:572027. doi: 10.3389/fpls.2020.572027 (PMC7667048; doi:10.3389/fpls.2020.572027)
Supplement: Supplementary file 1 [file Data_Sheet_1.docx]

**Supplementary Table S1:** Mean and variance components for GLS disease severity in each location for IMAS AM panel and biparental populations

| IMAS AM panel | Mean | σ^2^_G_ | σ^2^_e_ | h^2^ | LSD | CV |
| --- | --- | --- | --- | --- | --- | --- |
| Kitale2013 | 3.70 | 0.21* | 0.17 | 0.71 | 0.50 | 22.36 |
| Kitale2014 | 3.80 | 0.14* | 0.26 | 0.52 | 0.52 | 26.73 |
| Kakamega2013 | 4.30 | 0.14* | 0.30 | 0.49 | 0.55 | 25.45 |
| Kakamega2014 | 4.08 | 0.03* | 0.05 | 0.52 | 0.24 | 11.36 |
| CML550XCML494 DHpop1 | | | | | | |
| Kitale2015 | 4.60 | 0.04* | 0.20 | 0.29 | 0.35 | 19.39 |
| Kakamega2015 | 6.81 | 0.20* | 0.26 | 0.61 | 0.59 | 12.91 |
| CML550XCML504 DHpop2 | | | | | | |
| Kitale2014 | 3.66 | 0.02 | 0.18 | 0.20 | 0.27 | 23.48 |
| Kitale2015 | 3.54 | 0.05* | 0.30 | 0.26 | 0.40 | 31.06 |
| Kakamega2014 | 3.94 | 0.18* | 0.22 | 0.62 | 0.54 | 23.71 |
| Kakamega2015 | 5.26 | 0.09* | 0.10 | 0.66 | 0.38 | 11.97 |
| CML550XCML511 DHpop3 | | | | | | |
| Kitale2014 | 3.30 | 0.06* | 0.23 | 0.33 | 0.39 | 28.97 |
| Kitale2015 | 4.62 | 0.02 | 0.16 | 0.16 | 0.23 | 17.52 |
| Kakamega2014 | 4.04 | 0.04* | 0.35 | 0.19 | 0.36 | 29.15 |
| Kakamega2015 | 3.98 | 0.21* | 0.21 | 0.67 | 0.55 | 22.95 |
| CZL0618xLaPostaSeqC7-F71-1-2-1-1B F3pop4 | | | | | | |
| Kakamega2011 | 3.62 | 0.06* | 0.23 | 0.35 | 0.40 | 26.07 |
| Embu 2011 | 3.70 | 0.07* | 0.12 | 0.53 | 0.37 | 18.88 |
| CZL074xLaPostaSeqC7-F103-1-2-1-1B F3pop5 | | | | | | |
| Kakamega2011 | 4.05 | 0.04 | 0.28 | 0.20 | 0.34 | 26.24 |
| Embu 2011 | 3.32 | 0.02 | 0.09 | 0.30 | 0.17 | 18.22 |

* P = 0.05

**Supplementary Table S2:** Phenotypic correlations among locations for GLS resistance in IMAS AM panel and three DH populations.

| IMAS AM panel | Kitale2014 | Kitale2013 | Kakamega2013 |
| --- | --- | --- | --- |
| Kitale2013 | 0.90* |  |  |
| Kakamega2013 | 0.43* | 0.31* |  |
| Kakamega2014 | 0.38* | 0.40* | 0.60* |
| CML550XCML494 DHpop1 | Kitale2015 | Kitale2014 | Kakamega2015 |
| Kitale2014 | NA |  |  |
| Kakamega2014 | NA | NA |  |
| Kakamega2015 | 0.63* | NA | NA |
| CML550XCML504 DHpop2 | Kitale2015 | Kitale2014 | Kakamega2015 |
| Kitale2014 | 0.63* |  |  |
| Kakamega2014 | 0.59* | 0.82* |  |
| Kakamega2015 | 0.76* | 0.91* | 0.89* |
| CML550XCML504 DHpop3 | Kitale2015 | Kitale2014 | Kakamega2015 |
| Kitale2014 | 0.68* |  |  |
| Kakamega2014 | 0.52* | 0.91* |  |
| Kakamega2015 | 0.76* | 0.60* | 0.95* |

* P = 0.05

**Supplementary Table S3**. Summary of the linkage groups constructed based on data from three DH and two F3 populations.

| **Population** | **No. of environments** | **No. of progenies** | **No. of SNPs** | **Map length** | **Avg distance (cM)** |
| --- | --- | --- | --- | --- | --- |
| CML550 X CML494 - DH pop1 | 2 | 107 | 2105 | 3694.08 | 1.75 |
| CML550 X CML504 - DH pop2 | 4 | 211 | 2699 | 3896.04 | 1.44 |
| CML550 X CML511 - DH pop3 | 4 | 107 | 1962 | 3873.05 | 1.97 |
| CZL618 X LaPostaSeqC7-F71-1-2-1-1 -F3pop4 | 2 | 183 | 1130 | 4605.2 | 4.07 |
| CZL074 X LaPostaSeqC7-F103-1-2-1-1 -F3pop5 | 2 | 174 | 1047 | 4390.8 | 4.19 |


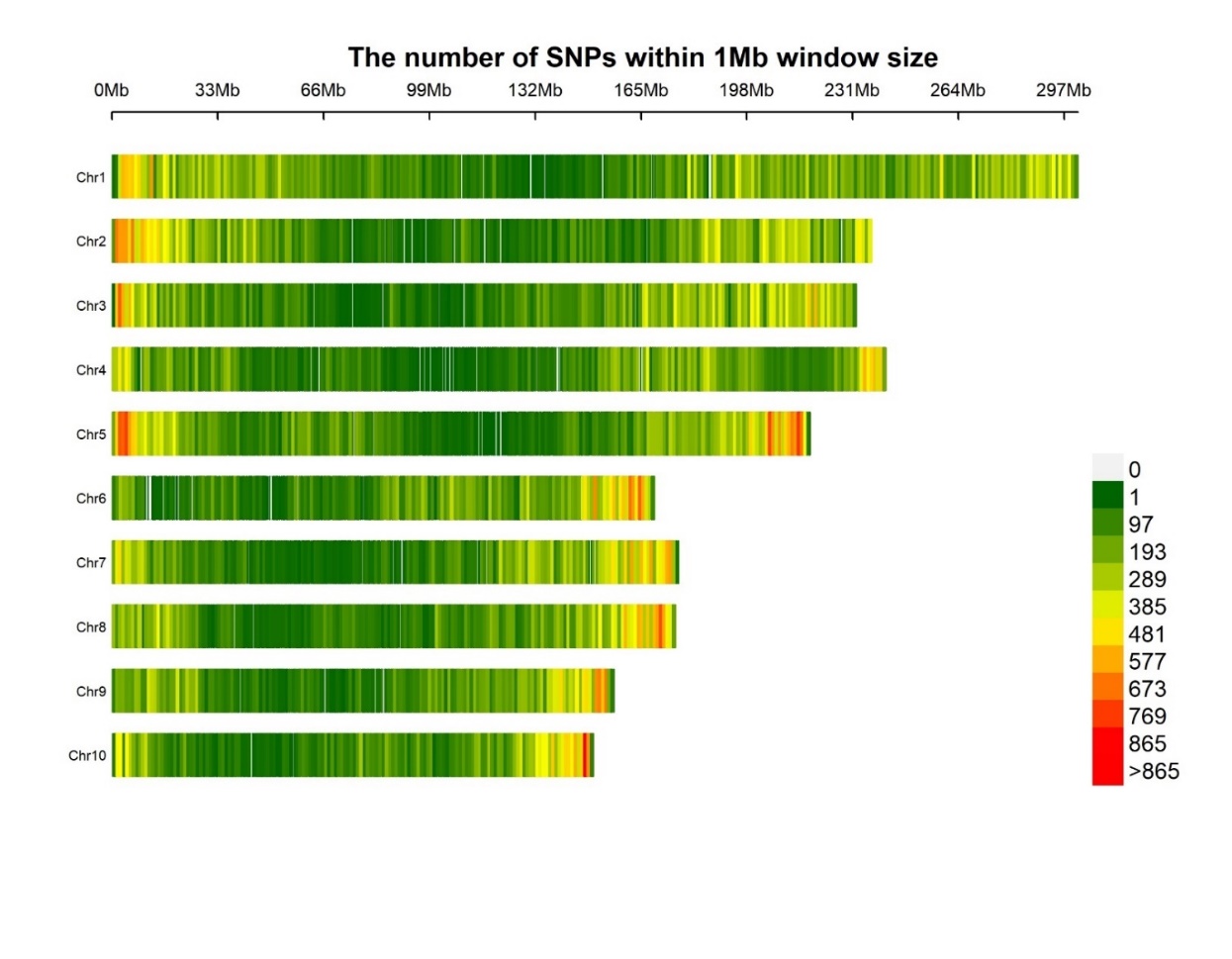


**Supplementary Figure S1**. Distribution of 337,148 GBS markers in the maize genome. The color key with marker densities indicates the number of markers within a window size of 1 Mb.

| 1 | 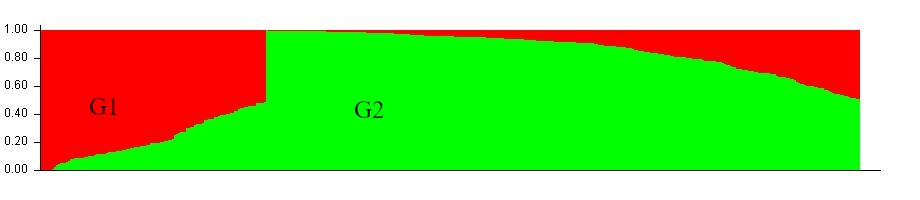 |
| --- | --- |
|  | 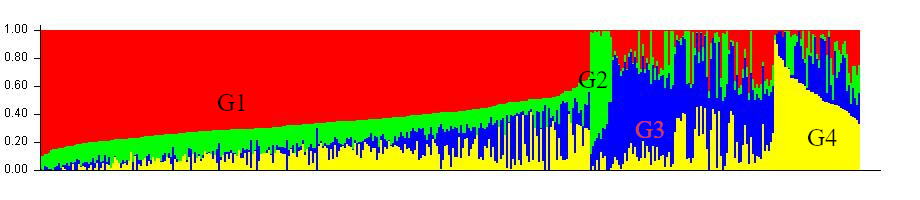 |
| 2 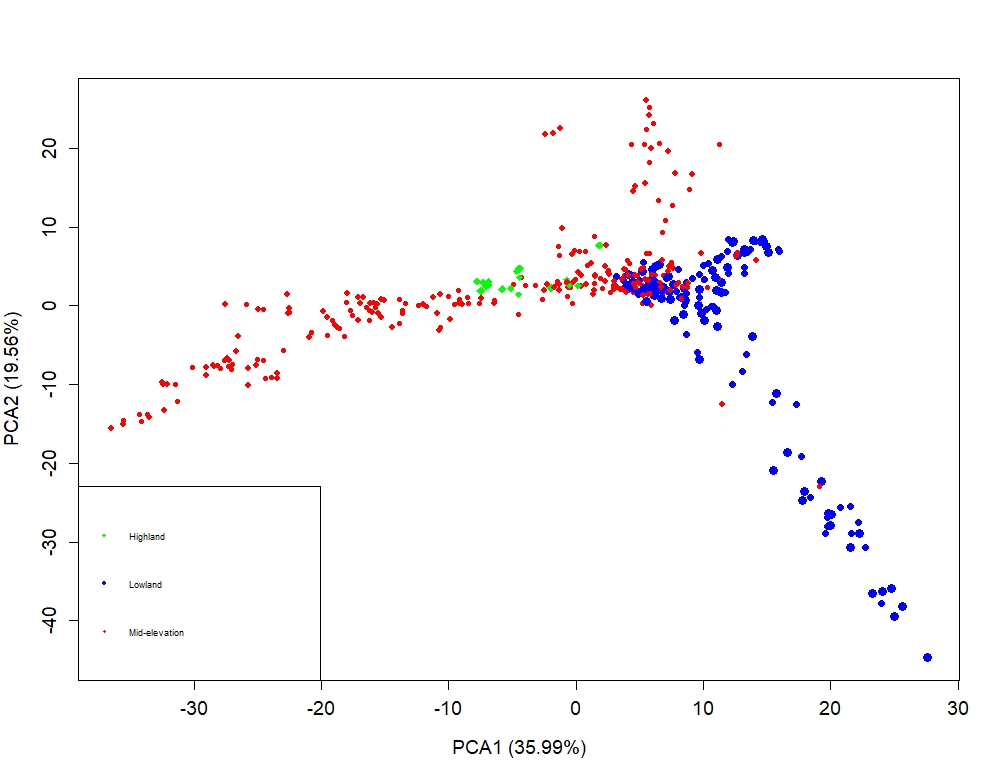 | |

**Supplementary Figure S2.** 1) Estimated population structure in the IMAS AM panel with of 410 maize lines, as revealed by 5061 polymorphic SNP markers. A) Estimation of the best number of population (K) from an assumed range of 1-2 based on evanno method. B) Graphical representation of population structure for peaks at K=2 and K=4. 2) Population structure based on principal component (PC) analysis of IMAS panel.


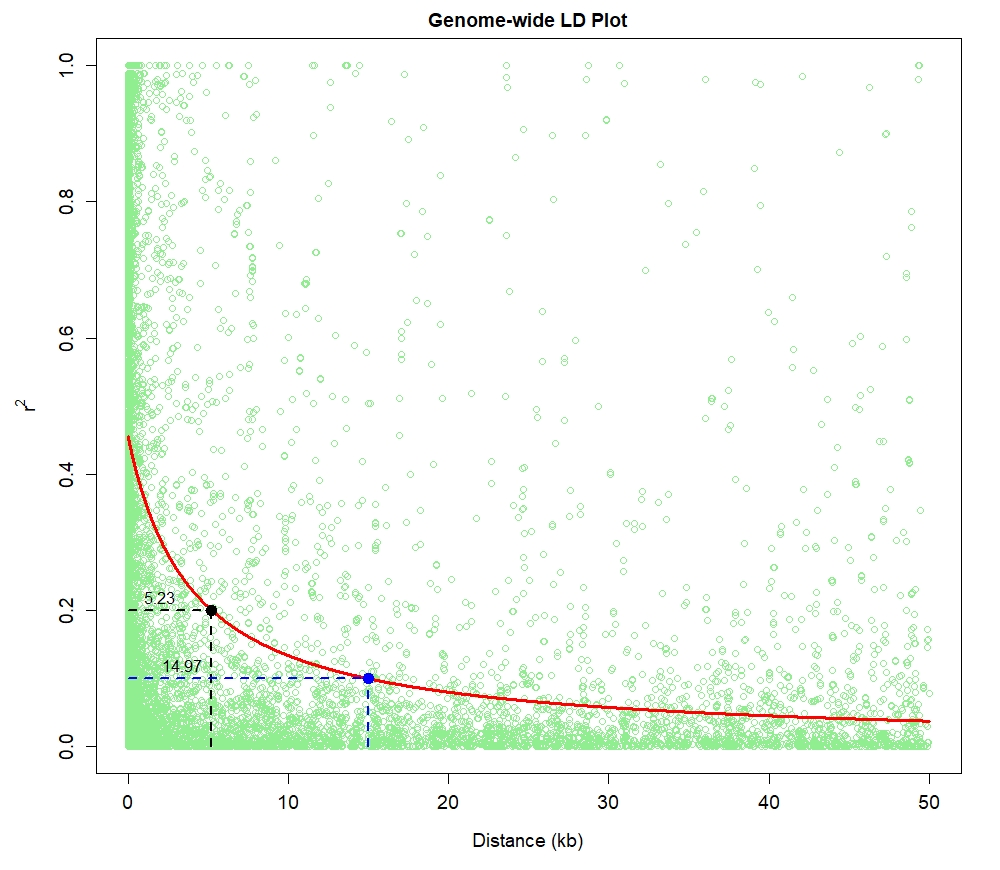


**Supplementary Figure S3.** Linkage disequilibrium plot representing the average genome wide LD decay within the IMAS panel with SNPs covering the whole genome. Y axis represents the squared correlation coefficient r^2^ while the X axis represents the physical distance on the chromosome.


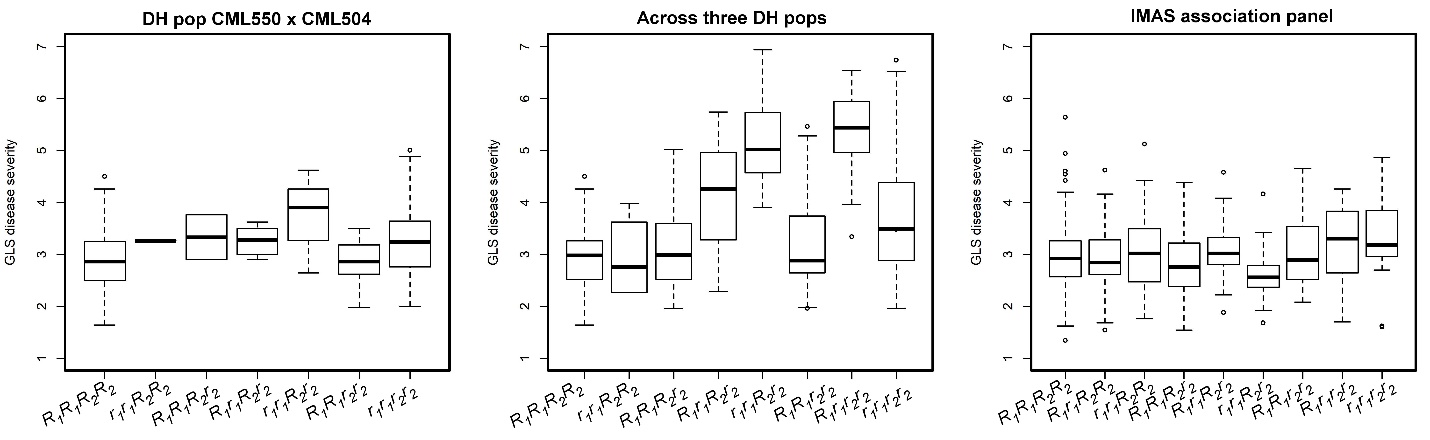


**Supplementary Figure S4.** Major QTL (qGLS7-105) for GLS resistance in the DH populations. Box–whisker plots display the level of disease resistance or severity for different allele combinations at resistance gene loci explaining > 20% of the phenotypic variation for GLS as determined by two strongly associated SNP markers
